# The Prognostic Value of the MiR-200 Family in Colorectal Cancer: A Meta-analysis with 1882 Patients

**DOI:** 10.7150/jca.27529

**Published:** 2019-07-08

**Authors:** Chengpeng Yu, Haiting Wan, Renfeng Shan, Wu Wen, Jianfeng Li, Daya Luo, Renhua Wan

**Affiliations:** 1 Department of General Surgery, The First Affiliated Hospital of Nanchang University, Nanchang, China.; 2 Department of Biochemistry and Molecular Biology, School of Basic Medical Sciences, Nanchang University, Nanchang, China

**Keywords:** MiR-200 family, Colorectal cancer, Prognostic Value, Meta-analysis

## Abstract

**Background**: MicroRNAs are small non-coding RNAs containing 18-22 nucleotides which play a role in RNA silencing and post-transcriptional regulation of their target genes. The MiR-200 family comprises miR-141, miR-200a, miR-200b, miR-200c and miR-429. Increasing evidence indicates that miR-200 microRNAs play a role in cancer metastasis. For example, miR-200 microRNAs were reported to influence the prognosis in colorectal cancer patients by regulating the expression of genes related to the epithelial-mesenchymal transition^6^. Previous studies have shown that the high expression of miR-200 microRNAs has an impact on the overall survival and Relapse-free Survival of CRC patients. However, the study results were inconsistent.

**Results**: Data from a total of 1882 patients from 9 studies was included in the meta-analysis. Poorer Relapse-free Survival (RFS) was observed in patients with high expression levels of miR-200 microRNAs (HR=1.13, 95% CI 1.04-1.23). Additionally, subgroup analysis of sample types revealed a significant association between higher expression of the miR-200 family in the plasma and poorer OS (HR=1.23, 95% CI 1.08-1.41) and RFS (HR=2.39, 95% CI 1.20-4.77), which indicates that the miR-200 family can be used as an easily detectable biomarker for evaluation of the prognosis of patients with colorectal cancer.

**Conclusions**: High expression levels of miR-200 microRNAs were associated with poor clinical outcomes in colorectal cancer patients. The miR-200 family can therefore potentially serve as a prognostic biomarker. Further studies should be performed to verify the clinical utility of the miR-200 family in colorectal cancer.

## Introduction

Colorectal cancer (CRC) is one of the most common cancers, responsible for 715,000 worldwide deaths in 2010 1. It is the second most common cause of cancer death in the USA 2. In China, colorectal cancer is the fourth most common cause of death and the fifth most common cancer type 3. In spite of the development of novel treatment options and early screening, the prognosis of CRC patients has not improved markedly. Furthermore, the incidence of CRC in China has been rising rapidly in recent years 4. Hence, novel biomarkers with clinical value are urgently needed to improve the compliance rate.

MicroRNAs are small non-coding RNAs containing 18-22 nucleotides which play a role in RNA silencing and post-transcriptional regulation of targeted genes. The miR-200 family contains miR-141, miR-200a, miR-200b, miR-200c and miR-429. Increasing evidence suggests miR-200 microRNAs played a role in cancer metastasis 5-14. In CRC, miR-200 microRNAs were reported to influence the prognosis of cancer patients by regulating genes related to the epithelial to mesenchymal transition 10, 15-19. Previous studies have shown that the high expression of miR-200 microRNAs has an impact on the overall- and Relapse-free Survival of CRC patients. However, the results were inconsistent. This paper aims to investigate the prognostic value of the MiR-200 family in patients with colorectal cancer via a meta-analysis of available literature.

## Materials and Methods

### Literature search

A comprehensive literature search was conducted in PMC (PubMed Central), PubMed, Web of Science, Embase, and Cochrane library, with a cut-off date of May 11, 2018, to obtain potentially eligible studies. The keywords for the search were (“miR-141” OR “miR-200a” OR “miR-200b” OR “miR-200c” OR “miR-429”) AND (“colorectal cancer” OR “Colorectal Tumor” OR “Colorectal Neoplasm” OR “Colorectal Carcinoma”) AND (“prognosis” OR “survival”). Additionally, other relevant articles were also obtained by manually screening the reference lists.

### Inclusion and exclusion criteria

Inclusion criteria for the articles were as follows: (1) the association between miR-200 family expression and overall survival (OS)/Relapse-free Survival (RFS) was investigated in patients with colorectal cancer; (2) the studies provided hazard ratios (HRs) and their 95% CIs of OS or RFS; (3) the patients were divided into low- and high expression groups according to the expression level of miR-200 microRNAs. Exclusion criteria for the articles were as follows: (1) duplicate publications; (2) studies with unusable data; and (3) reviews, letters, case reports and expert opinions.

### Data extraction and quality assessment

Two investigators (H-W and R-S) collected the data and information from all included studies. The following information was extracted from each study: first author name, publication year, total number of patients, outcome measures, the criteria for high miR-200 microRNA expression, determination method, hazard ratios (HRs) and corresponding 95% CIs. For all included studies, the results of multivariate analysis were only selected because of its increased precision for interpreting confounding factors. If a study provided the results of both multivariate and univariate outcomes, we chose the former. If a study only provided Kaplan-Meier survival curves, we extracted the corresponding survival data by using Engauge Digitizer V4.1 20. In the event of a disagreement, a third investigator (W-W) was consulted to reach a consensus. The quality of all included studies was assessed by using the Newcastle-Ottawa Scale (NOS) (http://www.ohri.ca/programs/clinical_epidemiology/oxford.asp). The NOS scores ranged from 0 to 9 and studies with NOS scores ≥ 6 were considered to be of high quality. The quality of all included studies varied from 7 to 9, with a mean value of 7.77 (Table 2).

### Statistical methods

The meta-analysis was conducted by using Stata SE12.0 (Stata Corp, College Station, TX) and R software 3.5.3 (Microsoft Revolution Analytics). The heterogeneity across studies was assessed by using the Chi square-based Q test and I^2^ statistics 21. A *P*-value of less than 0.05 for the Q test or an I^2^ value of more than 50 % was considered to indicate a significant difference. If the heterogeneity across studies was small (P_h_ > 0.05, I^2^ < 50%), the fixed effects model was adopted. Otherwise, the random effects model was applied (P_h_ ≤ 0.05, I^2^ ≥ 50%). Potential publication bias was calculated using Begg's test and Egger's test 22. A sensitivity analysis was also conducted to evaluate the stability of the results. Differences with *P*-values of less than 0.05 were considered statistically significant.

## Results

### Study characteristics

**Figure 1:**
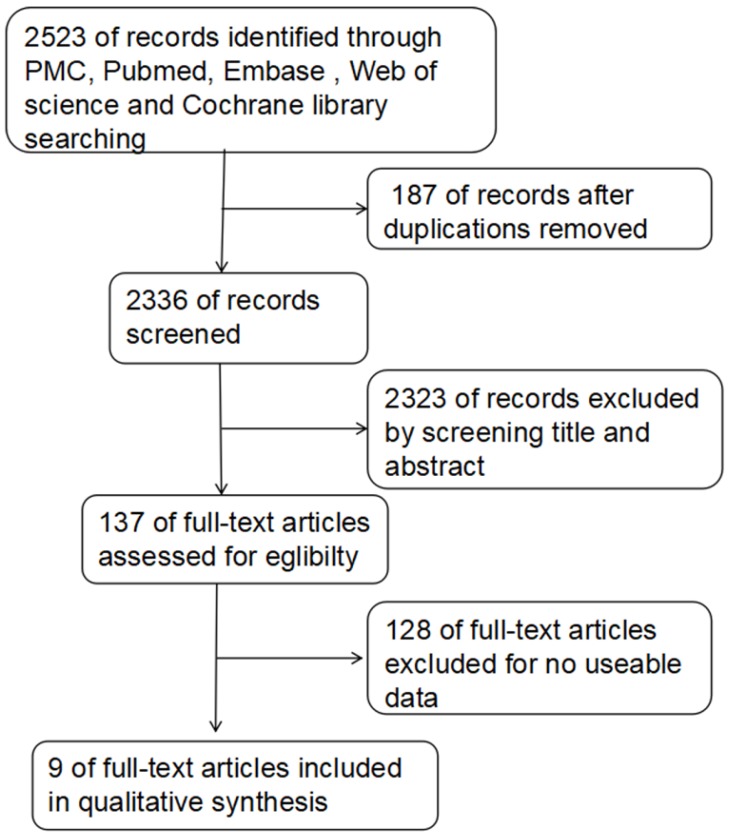
Flowchart presenting the steps of literature search and selection.

The detailed process of literature retrieval is shown in Figure 1. A total of 1882 cancer patients in 9 studies 23-31 were ultimately included in the meta-analysis. The mean sample size was 209 patients (from 78 to 543). The included studies were from America, Asia and Europe (Table 1).

### The expression of miR-200 microRNAs and overall survival

**Figure 2:**
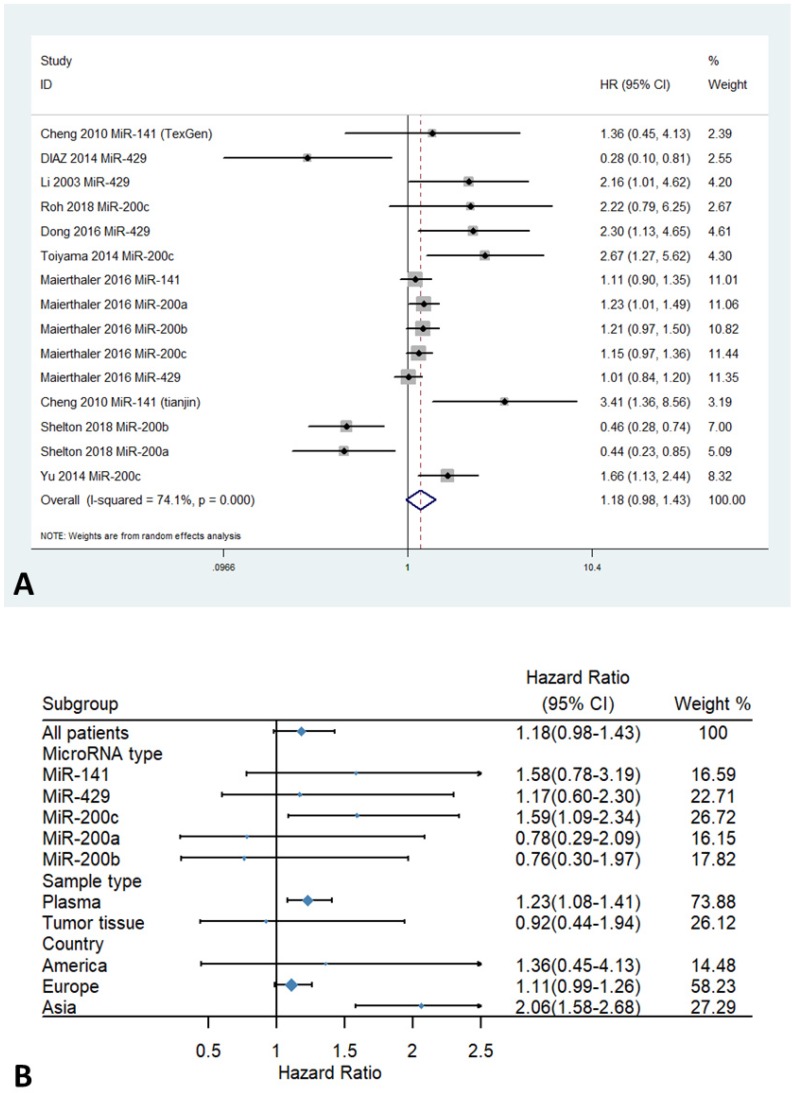
Forest plots of HR for the relationship between high miR-200 microRNA expression and OS: (A) overall, (B) stratified by country, (C) stratified by miRNA type, (D) stratified by sample type.

The forest plots for the association between miR-200 expression and overall survival of patients with colorectal cancer are shown in Figure 2. Overall, The HRs of the high miR-200 microRNAs expression group versus the low miR-200 microRNAs expression group was 1.18 (95% CI 0.98-1.43, Figure 2A). Stratified analysis by sample types showed a significant association between enhanced expression of miR-200c in plasma and poor OS (HR=1.23, 95% CI 1.08-1.41, Figure 2B). Moreover, a significant association was found between higher expression of the miR-200 family and poorer OS in the Asian population (HR=2.06, 95% CI 1.58-2.68), compared with the European population (HR=1.11, 95% CI 0.99-1.26, Figure 2B) (Table 3).

### The expression of miR-200 microRNAs and Relapse-free Survival

**Figure 3:**
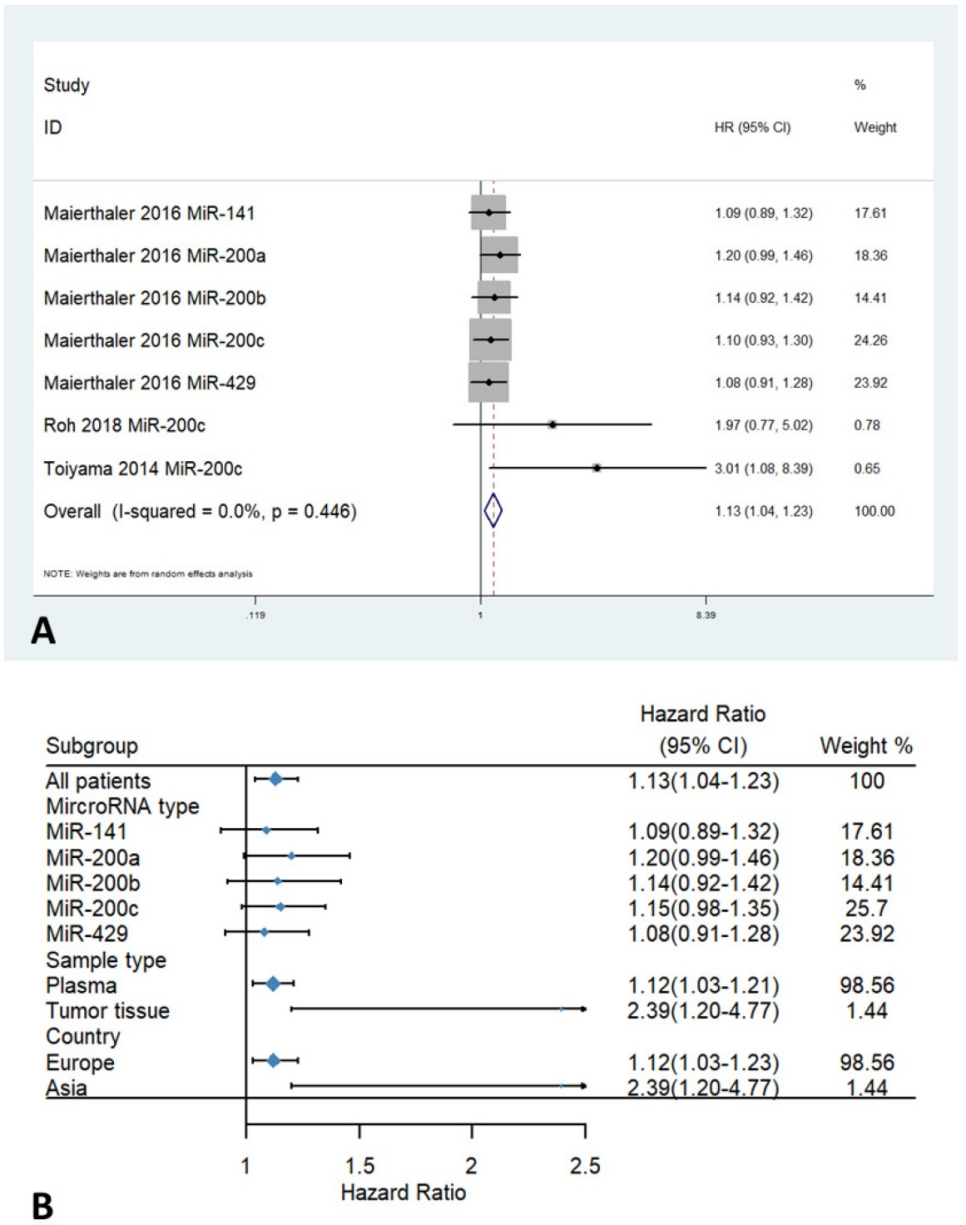
Forest plots of HR for the relationship between high miR-200 microRNA expression and RFS: (A) overall, (B) stratified by country, (C) stratified by miRNA type, (D) stratified by sample type.

Only three studies with a total of 1098 patients contained data on Relapse-free Survival (RFS). No obvious statistical heterogeneity was observed among the included studies (I^2^ = 0 %; P_h_ = 0.446). The fixed-effects model was adopted to assess the pooled hazard ratios (HRs) and corresponding 95% confidence intervals (CIs). The overall result demonstrated a strong association between high expression levels of miR-200 microRNAs and poor RFS (HR=1.13, 95% CI 1.04-1.23, Figure 3A). Stratified analysis by country revealed a poorer RFS in patients with high expression levels of miR-200 microRNAs from Asia. Furthermore, stratified analysis by sample types found a significant association between higher expression of the miR-200 family and poor RFS in plasma (HR= 1.12, 95% CI 1.03-1.21, Figure 3B) (Table 3).

### Sensitivity analysis

**Figure 4:**
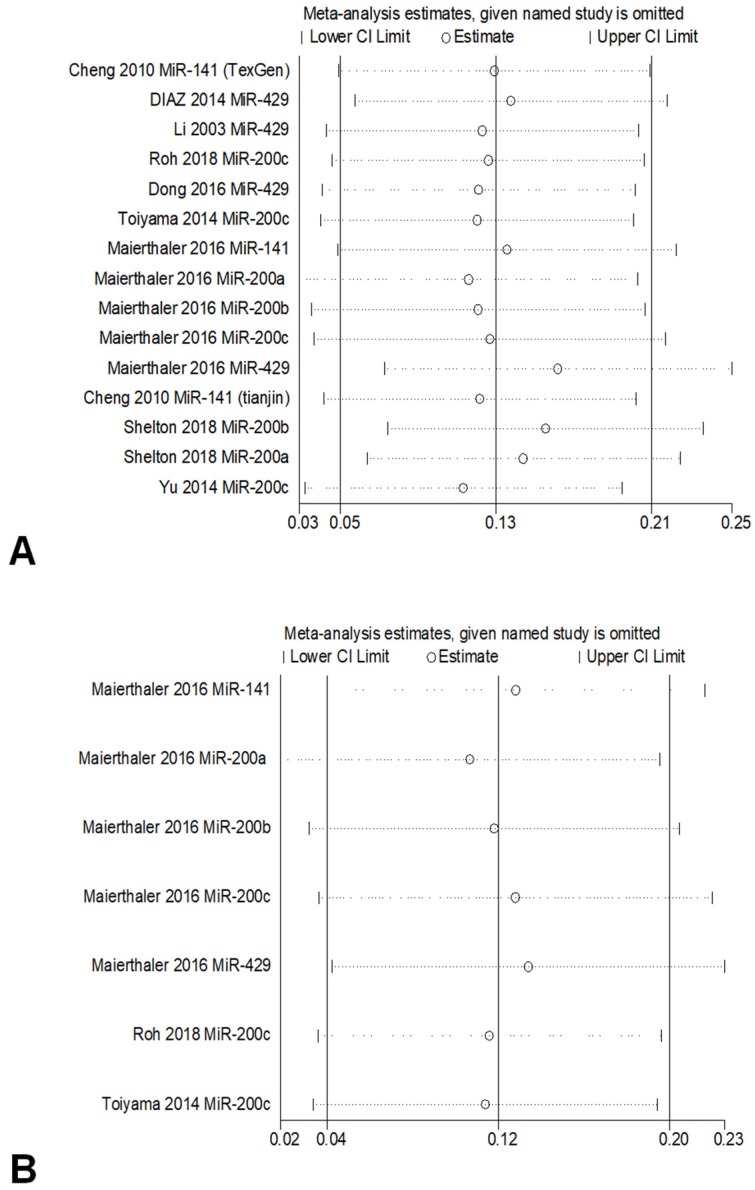
Sensitivity analyses of studies regarding overall survival (a) and Relapse-free Survival (b).

To investigate the correlation between the expression of miR-200 microRNAs and OS, sensitivity analysis was conducted by sequentially omitting each study from the pooled analysis, one at a time. The result was not obviously influenced by the exclusion of any study, which underscored the robustness of the results (Figure 4).

### Publication bias

**Figure 5:**
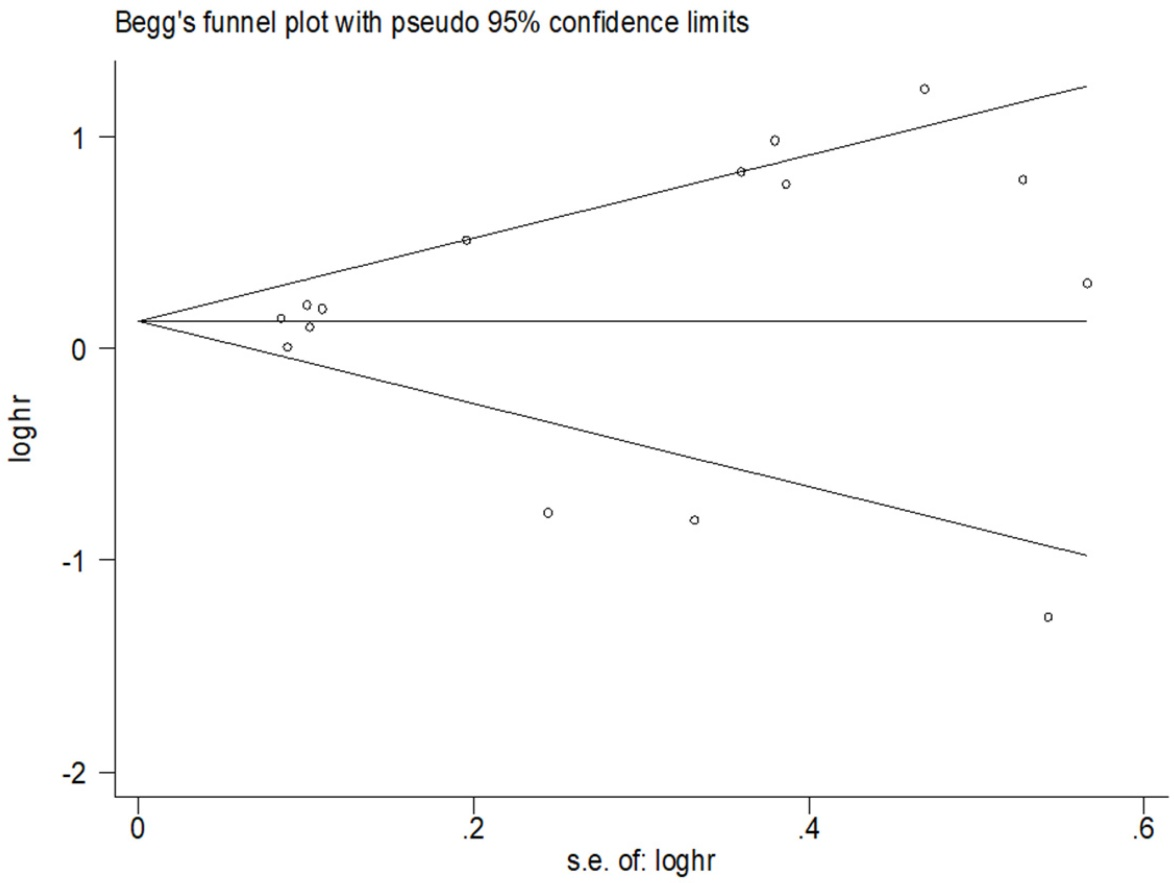
Results of publication bias for OS.

For the relationship between miR-200 microRNA expression levels and OS, Begg's test and Egger's test were applied to test for publication bias. No publication bias was observed in the included studies (Figure 5).

## Discussion

MiR-200 microRNAs were found to act together with ZEB1 and ZEB2 proteins to regulate the epithelial-mesenchymal transition (EMT) and mesenchymal-epithelial transition (MET) in the development of CRC and influence its prognosis^15^, ^32^. Some studies showed that high expression of MiR-200 microRNAs was associated with improved OS in CRC patients 15, 29. However, other studies demonstrated that the high expression of MiR-200 microRNAs was related to poor OS in comparable patients 24-28. Although the inconsistent results of these studies have not yet been explained in detail, the discrepancy might partly attributed to the different results of microarray analysis, since these miRNA profiling studies were conducted with different microarray platforms. Furthermore, small differences of experimental design across studies could also affect the observed results. Notably, the insufficient number of tumor samples in the inclusive studies could have led to limited statistical power, diminishing the observed prognostic value of the miR-200 family.

In the current paper, we assessed the relationship between the survival of patients with colorectal cancer and miR-200 expression. The pool analysis showed that higher expression of miR-200 family RNAs was observed to be related to poor RFS in patients with colorectal cancer. Furthermore, the subgroup analysis of sample types found a significant association between higher expression of the miR-200 family in the ser poorer OS and RFS, which implied that the miR-200 family might become an easily detectable biomarker for the evaluation of the prognosis of patients with colorectal cancer.

However, there are some limitations to our meta-analysis. Firstly, the total sample size was relatively small and we failed to detect a relationship between the expression of miR-200 microRNAs and clinicopathological parameters. Secondly, there was significant heterogeneity across studies regarding the association of the miR-200 family with OS. Subgroup analysis demonstrated that the country of origin of the inclusive studies might be the main source of heterogeneity. Thirdly, the number of inclusive studies is small, whose statistical power is still limited. Although the result of publication bias demonstrated that there did not exist publication bias, there may exist publication bias because of limited inclusive studies. Fourthly, there still exists small differences in the role of each member of miR-200 family in colorectal cancer, which may cause also affect the observed results. Fifth, estimating HRs from Kaplan Meier curves can become inaccurate when there are very small differences in overall OS and RFS, which may also have impacted the results of this meta-analysis. Furthermore, the different methods/platforms used for mRNA detection, inconsistent definition of OS/RFS, various sample sources and different types of disease might cause a deviation of the results of this meta-analysis. Finally, this paper did not assess the prognostic value of a combination of miR-200 and other miRNA markers. Therefore, larger-size, multi-center and higher-quality studies with a unified criterion for determining the expression of miR-200 family microRNAs are necessary to validate the results in this study.

## Figures and Tables

**Table 1:** Characteristics of studies included in the meta-analysis.

Study	Year	Sample size	Country	RNA	HR (OS)	LCI (OS)	UCI (OS)	HR (RFS)	LCI (RFS)	UCI (RFS)	Sample type
Cheng	2010	102	USA	miR-141	1.36	0.45	4.14				plasma
Cheng	2010	156	China	miR-141	3.41	1.36	8.56				plasma
DIAZ	2014	127	Spain	miR-429	0.28	0.05	0.42				tumor tissues
Dong	2016	78	China	miR-429	2.296	1.10	4.52				tumor tissues
Li	2013	110	China	miR-429	2.165	1.015	4.618				tumor tissues
Maierthaler	2016	543	Germany	miR-141	1.105	0.904	1.35	1.085	0.89	1.321	plasma
Maierthaler	2016	543	Germany	miR-200a	1.227	1.008	1.495	1.2	0.989	1.456	plasma
Maierthaler	2016	543	Germany	miR-200b	1.208	0.975	1.497	1.143	0.919	1.422	plasma
Maierthaler	2016	543	Germany	miR-200c	1.152	0.975	1.362	1.1	0.93	1.302	plasma
Maierthaler	2016	543	Germany	miR-429	1.006	0.845	1.198	1.078	0.91	1.277	plasma
Roh	2018	109	Korea	miR-200c	2.22	0.79	6.26	1.96	0.77	5.02	tumor tissues
Toiyama	2014	446	Japan	miR-200c	2.67	1.28	5.67	3.01	1.08	8.39	plasma
Yu	2014	103	China	miR-200c	1.665	1.13	1.135				plasma
Shelton	2018	108	USA	miR-200b	0.459	0.284	0.74				tumor tissues
Shelton	2018	106	USA	miR-200a	0.444	0.216	0.793				tumor tissues

**Table 2:** Methodological quality of studies included in the meta-analysis.

Study	Cheng	DIAZ	Dong	Li	Maierthaler	Roh	Toiyama	Yu	Shelton
Representativeness of the exposed cohort	☆	☆	☆	☆	☆	☆	☆	☆	☆
Selection of the non-exposed cohort	☆	☆	☆	☆	☆	☆	☆	☆	☆
Ascertainment of exposure	☆	☆	☆	☆	☆	☆	☆	☆	☆
Demonstration that outcome of interest was not present at start of study	☆	☆	☆	☆	☆	☆	☆	☆	☆
Comparability of cohorts on the basis of the design or analysis	☆☆	☆	☆☆	☆☆	☆	☆	☆☆	☆	☆
Assessment of outcome	☆	☆	☆	☆	☆	☆	☆	☆	☆
Was follow-up long enough for outcomes to occur	-	☆	-	☆	☆	☆	-	-	☆
Adequacy of follow-up of cohorts	-	☆	-	☆	☆	☆	-	☆	-
Stars	7	8	7	9	8	8	7	8	8

**Table 3:** Results of quantitative analysis

Subgroup	HR	95% CI	I^2^ (%)	P_H_
OS				
Overall	1.18	0.98-1.43	74.1	<0.001
Member type				
miR-141	1.58	0.78-3.19	64.1	0.061
miR-200a	0.78	0.29-2.09	88.4	0.003
miR-200b	0.76	0.30-1.79	92.3	<0.001
miR-200c	1.59	1.09-2.34	63.3	0.043
miR-429	1.17	0.60-2.30	79.1	0.002
Location				
Asia	2.06	1.58-2.68	0	0.71
Europe	1.11	0.99-1.26	46.8	0.094
America	0.56	0.33-0.91	40.5	0.186
Sample type				
Plasma	1.23	1.08-1.41	52.3	0.033
Tumor tissues	0.92	0.44-1.94	83.9	<0.001
RFS				
Overall	1.13	1.04-1.23	0	0.446
Member type				
miR-200c	1.15	0.98-1.35	59.4	0.085
Location				
Asia	2.39	1.20-4.77	0	0.549
Europe	1.12	1.03-1.21	0	0.931
Sample type				
Plasma	1.12	1.03-1.21	0	0.549
Tumor tissues	2.39	1.20-4.77	0	0.931
